# Prevalence of Over-the-Counter and Prescription Medication Use in the US

**DOI:** 10.1001/jamanetworkopen.2025.59479

**Published:** 2026-02-16

**Authors:** Jody L. Green, Taryn Dailey-Govoni, Sita D. Kalidindi, Suzanne K. Vosburg

**Affiliations:** 1 Uprise Health, Irvine, California; 2 Ephicacy Lifescience Analytics, Bangalore, India

## Abstract

**Importance:**

In the US, reliance on over-the-counter (OTC) and prescription medications has important public health implications. Understanding the prevalence of medication use, overall and by specific medication, aids in regulatory decision-making and pharmacoeconomic evaluations and informs measures of benefits and risks.

**Objective:**

To estimate the current prevalence of OTC and prescription medication use in the US adult population.

**Design, Setting, and Participants:**

This survey study analyzed data collected via a population-based, online survey of the US adult population (June 2023 to April 2024). Participants were volunteer online panelists aged 18 years and older.

**Exposure:**

Use of any OTC or prescription medication.

**Main Outcomes and Measures:**

The primary outcome was prevalence of OTC and prescription medication use (overall and by specific medication), stratified by sex and age. Prevalence estimates were generated by calculating the proportion of the population reporting the key outcomes.

**Results:**

Of the 21 000 participants, 11 171 (53.2%) were female, and the mean (SD) age was 47.9 (17.5) years. Past-7-day prevalence of OTC or prescription medication use was 62.3% (13 073 individuals). The use of 5 or more medications was reported by 3425 participants (16.3%), and 690 (3.3%) reported taking 10 or more medications. Although use was higher among female individuals (7442 participants [66.6%]) than among male individuals (5631 participants [57.3%]), the patterns of increasing use and increasing number of medications with age were similar. The past-7-day prevalence of OTC medication use (9657 participants [46.0%]) was similar to that of prescription medications (9719 participants [46.3%]). Acetaminophen (6184 participants [29.4%]), ibuprofen (4693 participants [22.3%]), and aspirin (3323 participants [15.8%]) were the most reported medications used in the past 7 days. Six of the 10 most prevalent medications reported were available OTC. The most reported prescription medications were atorvastatin (1342 participants [6.4%]), lisinopril (1163 participants [5.5%]), levothyroxine (1086 participants [5.2%]), and amlodipine (965 participants [4.6%]).

**Conclusions and Relevance:**

In this 2023 to 2024 study, nearly 2 of 3 US adults reported medication use in the past 7 days. Past-7-day prevalence of OTC and prescription medication use was similar, demonstrating the reliance on these therapies and highlighting the importance of accessibility. Medication-specific prevalence allows for a better understanding of actual use and the ability to estimate potential benefits and risks associated with medication access or regulatory changes. Continued monitoring is necessary to measure the benefits and risks of regulatory decisions or other market changes that may affect medication access or use.

## Key Points

**Question:**

What is the current prevalence of over-the-counter (OTC) and prescription medication use in the US?

**Findings:**

In this survey study of 21 000 US adults, 62.3% reported past-7-day use of any OTC or prescription medication. Past-7-day prevalence of any OTC medication use (46.0%) was similar to that of any prescription medication use (46.3%) and illustrated a similar pattern of increased use with age among both male and female individuals.

**Meaning:**

The high prevalence of OTC and prescription medication use highlights the reliance on these products, aids in regulatory decision-making, contributes to pharmacoeconomic evaluations, and informs measures of benefits and risks.

## Teaser

This survey study estimates the current prevalence of over-the-counter and prescription medication use in the US adult population.

## Introduction

Over-the-counter (OTC) and prescription medications are vital to everyday living for many people, with 81% of adults in the US using at least 1 OTC medication, prescription medication, or vitamin, mineral, or herbal supplement in the past week^1^ and 64.8% of US adults taking at least 1 prescription medication annually.^2^ In 2024, OTC medication sales were estimated at $44.3 billion,^3^ and the Centers for Medicare & Medicaid Services estimated prescription drug expenditure exceeded $463 billion.^4^ Despite this widespread use, there is no ongoing data source that monitors prevalence of actual medication usage. Point-of-sale data for OTC medications and pharmacy dispensing records for prescription medications are useful in understanding the types and amounts of medications obtained by patients but do not measure actual usage.

The most recent data examining the prevalence of use at a medication-specific level are from the Slone Survey,^5^ which included 2590 telephone surveys collected via random-digit-dialing of an adult noninstitutionalized population from February 1998 through December 1999. In that study, 81% of respondents reported past-week use of OTC or prescription medication, or a vitamin, mineral, or herbal supplement, and 50% reported past-week prescription medication use. Acetaminophen, ibuprofen, and aspirin were the most commonly reported.

Although the Slone Survey included prevalence of all medications in the US adult population, more recent studies have focused on use in older adults^6,7,8,9,10,11,12,13,14,15,16,17,18,19^ or on a limited array of medications (such as analgesics^20^ or OTC only^11,19,21^). The paucity of contemporary US data, particularly of medication-specific usage, may reflect the challenges with measuring medication use. First, data collection relies on self-reported historical information, which is subject to recall bias. Studies have reported 73% to 79% accuracy for past 30-day medication use by means of a structured medication history method.^22,23^ Accuracy of recall was higher for prescription medications than for OTC and was highest for more recent recall periods (approximately 80%-90% accuracy for medications taken the preceding day, approximately 80% for the preceding week, and approximately 65%-75% for the preceding 3 weeks). Second, various definitions have been used for usage (any medication use, use for specific purpose, or use of specific medications), time frames (lifetime, preceding 30 days, or preceding week), and users (individual vs household use) which prohibits cross-study comparisons. There are also challenges with medication and general health literacy, particularly with product identification. Finally, there may be seasonal variations, such as with allergy or cold and influenza products, which need to be accounted for in the study design.

The study of medication use is an important component of understanding the magnitude of associated health benefits and risks, particularly when considering policy and regulatory changes, as well as after introduction of a new medication. Therefore, the objective of this study was to estimate current prevalence of OTC and prescription medication use in the US adult population using contemporary study methods.

## Methods

### Study Design

This was a survey study of the general US population aged 18 years and older using an online survey regarding use of OTC and prescription medications. The WCG institutional review board approved this study on June 8, 2023. The study planning, design, data collection and analysis, and reporting followed the American Association for Public Opinion Research (AAPOR) reporting guideline best practices for survey research.

### Participants, Data Source, and Study Size

Participants were recruited by YouGov, a survey panel company.^24^ YouGov uses a validated sample-matching approach informed by data from large nationally representative surveys. Selection within strata is conducted by weighted sampling with replacements. Sample-matching methods have been shown to yield population-level estimates comparable to those obtained through random-digit–dial telephone surveys after appropriate weighting.^25^ Eligible individuals who provided electronic informed consent were directed to complete the survey and received compensation based on YouGov policies.^24^ Individuals without access to the internet or smartphone were not eligible to participate; however, sample matching minimizes resulting potential bias. Data collection was completed in 3 separate time frames to account for potential seasonality. YouGov uses measures to detect fraudulent, inconsistent, or invalid responses for removal.^26^

### Variables

Demographic information, including self-identified race and ethnicity and sex, was collected. Data on race and ethnicity were obtained to characterize the population and were necessary to apply the sampling frame. Medical history was collected by providing a list of health conditions and inquiring about lifetime history and current status. The collection of medication use was based on the published MedHAT method,^22,23^ using free recall and prompt-based recall to optimize accuracy. Participants were first instructed to list the medications they had used in the past 7 days (free recall and open text). They were shown sample OTC and prescription labels to illustrate where they could find the information requested, instructed to gather their medications and use the labels for reference, and encouraged to use the internet or other resources to find product information as needed. The product name and active ingredient fields had an autofill feature that offered suggested names as the information was entered. Participants were then presented with 3 prompts to further assist with medication recall: (1) common ailments, (2) self-reported medical history, and (3) past-7-day activities. Medication-specific questions were asked regarding the most recent use of acetaminophen, ibuprofen, aspirin, and naproxen, as these are the most common OTC medications and are available in a multitude of products.

The principal investigator (J.L.G.) reviewed each pair of medication name and active ingredient(s). Instances of misspellings or mismatches were reconciled. Vitamins, herbal supplements, and topical treatments were excluded. A confidence rating was generated for each medication to classify the quality of information and participants’ ability to follow instructions. The predefined confidence ratings included absolute confidence (exact match between medication name and active ingredients with correct spelling), high confidence (misspellings present but information interpretable with little doubt), moderate confidence (medication name did not match active ingredients and/or >1 drug was reported on the same line, requiring recoding), and low confidence (indecipherable report [gibberish] or reports of a type of medication [eg, hypertensive] without specifying any active ingredient). Low confidence entries were excluded.

A second investigator (T.D.-G.) conducted quality control by reviewing a 5% random sample of the medication data to confer the coding of medication name, active ingredients, prescription status, and confidence rating. An agreement rate of 95% or better was predetermined as the acceptable quality level. Additional systematic data edit checks were conducted to ensure consistency of coded data.

### Statistical Analysis

Descriptive statistics were used to summarize demographic information, medical history, and other categorical variables. Prevalence estimates were generated by calculating the proportion of the population reporting the key outcomes. The overall prevalence for any OTC and any prescription medication were calculated at the individual case level, regardless of the number of medications reported. For the medication-specific prevalence estimates, fixed-dose combination products were included in the counts for each medication. For example, report of a product that contained acetaminophen and diphenhydramine was included in the prevalence for both medications. All analyses were performed using SAS statistical software version 9.4 M3 (SAS Institute).

## Results

### Participants

A total of 25 052 surveys were completed. Of these, 2869 were excluded during the YouGov data cleaning process and 1183 were removed in the sample-weighting process, resulting in a final predetermined analytic sample of 21 000 participants (7000 completions per launch: summer [June 16, 2023, to July 12 2023], fall [October 11, 2023, to October 31, 2023], and spring [March 25, 2024, to April 9, 2024]). The mean (SD) age was 47.9 (17.5) years (Table 1). Female individuals accounted for 53.2% (11 171 participants), among whom 265 (2.4%) indicated a current pregnancy. With regard to race, 650 participants (3.1%) were American Indian or Alaska Native, 848 (4.0%) were Asian, 3081 (14.7%) were Black or African American, and 16 042 (76.4%) were White. Participants were mostly married and living with a spouse (9446 participants [45.0%]), whereas 6357 (30.3%) were never married, and 2302 (11.0%) were divorced. Participants mostly worked full-time (8228 participants [39.2%]) and 2978 participants (14.2%) worked part-time. Health care coverage was provided by employer insurance for 7956 participants (37.9%), by Medicare for 5020 participants (23.9%), and by Medicaid or other government assistance for 4602 participants (21.9%). Alcohol use in a typical week was reported by 9421 participants (44.9%), among whom the majority used alcohol 1 to 2 days per week. Lifetime use of tobacco was reported by 9547 participants (45.5%), of whom 8969 (93.9%) reported past-year use of cigarettes or cigars, 4018 (42.1%) past year use of e-cigarettes or vape pen, and 2210 (23.1%) past year use of smokeless tobacco. Of the top 30 medical conditions (Table 2), those most likely to have been treated with medication at the time of study participation were (percentage of those with the condition) hypertension (4610 participants [73.1%]), diabetes (1855 participants [71.2%]), anxiety or panic disorders (2702 participants [43.4%]), depression (2720 participants [40.7%]), and allergies (4406 participants [39.9%]).

**Table 1.** Survey Participant Demographics

Characteristic	Participants, No. (%)
Age, mean (SD), y	47.9 (17.5)
Age group, y	
18-24	2066 (9.8)
25-34	3841 (18.3)
35-44	3712 (17.7)
45-54	3207 (15.3)
55-64	3777 (18.0)
65-74	2976 (14.2)
75-84	1292 (6.2)
≥85	129 (0.6)
Sex	
Male	9829 (46.8)
Female	11 171 (53.2)
Current pregnancy among female individuals (n = 11 171)	
Yes	265 (2.4)
No	10 906 (97.6)
Ethnicity	
Not Hispanic, Latino/a, or Spanish	17 953 (85.5)
Hispanic, Latino/a, or Spanish	3047 (14.5)
Race (select all that apply)	
American Indian or Alaska Native	650 (3.1)
Asian	848 (4.0)
Black or African American	3081 (14.7)
Native Hawaiian or other Pacific Islander	127 (0.6)
White	16 042 (76.4)
Other^a^	1100 (5.2)
Marital status	
Married, living with spouse	9446 (45.0)
Separated	442 (2.1)
Divorced	2302 (11.0)
Widowed	1124 (5.4)
Never married	6357 (30.3)
Domestic partnership	1329 (6.3)
Highest level of education completed	
No high school degree	798 (3.8)
High school degree	7077 (33.7)
Some college or technical vocational school, but no degree (yet)	3347 (15.9)
Trade of vocational degree	813 (3.9)
2-y College degree	1687 (8.0)
4-y College degree	4607 (21.9)
Postgraduate degree	2671 (12.7)
Current enrollment in college or university or trade or vocational school	
Yes	1747 (8.6)
No	18 455 (91.4)
Current employment status	
Working full-time	8228 (39.2)
Working part-time	2978 (14.2)
Unemployed	2261 (10.8)
Military	31 (0.1)
Retired	4306 (20.5)
Disabled	1383 (6.6)
Caretaker, homemaker, or volunteer	1310 (6.2)
Other	503 (2.4)
Annual income, $	
<10 000	1519 (7.2)
10 000-19 999	1580 (7.5)
20 000-29 999	2123 (10.1)
30 000-39 999	1866 (8.9)
40 000-49 999	1754 (8.4)
50 000-74 999	3270 (15.6)
75 000-99 999	2544 (12.1)
100 000-149 999	2732 (13.0)
150 000-249 999	1255 (6.0)
≥250 000	530 (2.5)
Prefer not to answer	1827 (8.7)
Health care cost coverage (select all that apply)	
Insurance through a current or former employer or union (yours or another family member’s)	7956 (37.9)
Insurance purchased directly from an insurance company (by you or another family member)	2646 (12.6)
Medicare, for people aged ≥65 y, or people with certain disabilities	5020 (23.9)
Medicaid, Medical Assistance, or any kind of government-assistance plan for those with low incomes or a disability	4602 (21.9)
TRICARE or other military health care	544 (2.6)
VA (enrolled for VA health care)	579 (2.8)
Indian Health Service	107 (0.5)
Self-pay (no insurance or other coverage to help with health care costs)	2049 (9.8)
Any other type of health insurance or health coverage	711 (3.4)
During a typical week, how many days do you drink alcohol?	
Never	11 579 (55.1)
1-2 d/wk	6169 (29.4)
3-4 d/wk	1919 (9.1)
5-7 d/wk	1333 (6.3)
On the days you drink alcohol, how many drinks do you typically have? (among alcohol users, n = 9421)	
1	3419 (36.3)
2	3207 (34.0)
3	1448 (15.4)
4	681 (7.2)
≥5	666 (7.1)
Have you ever used any form of tobacco?	
Yes	9547 (45.5)
No	11 453 (54.5)
What kind of tobacco have you used in the past year? (select all that apply; Among lifetime tobacco users, n = 9547)	
Cigarettes, cigars, cigarillos, pipes	8969 (93.9)
Smokeless tobacco (eg, chew or snuff)	2210 (23.1)
E-cigarettes or vape pen	4018 (42.1)
None	23 (0.1)

Abbreviation: VA, Veterans Affairs.

^a^ Other was an open-text field for race identification where individual responses could be written in if participants felt their race was not adequately captured by the existing categories.

**Table 2.** Top 30 Lifetime Conditions and Current Status

Condition	Experienced symptoms, been diagnosed, or treated in your lifetime, No. (column %) (N = 21 000)	Participants, No. (row %)	
		Experiencing symptoms	Taking medication
High blood pressure (hypertension)	6309 (30.0)	889 (14.1)	4610 (73.1)
Diabetes	2605 (12.4)	563 (21.6)	1855 (71.2)
Anxiety or panic disorders	6220 (29.6)	2059 (33.1)	2702 (43.4)
Depression	6677 (31.8)	2097 (31.4)	2720 (40.7)
Allergies	11 041 (52.6)	2300 (20.8)	4406 (39.9)
Asthma	3682 (17.5)	698 (19.0)	1443 (39.2)
Arthritis (rheumatoid arthritis or osteoarthritis)	4636 (22.1)	1746 (37.7)	1659 (35.8)
Osteoporosis	1438 (6.8)	305 (21.2)	515 (35.8)
Heartburn or reflux	10 603 (50.5)	1432 (13.5)	3494 (33.0)
Degenerative disc disease (back disease, spinal stenosis, or severe chronic back pain)	2799 (13.3)	1132 (40.4)	907 (32.4)
Attention deficit disorder and/or attention-deficit/hyperactivity disorder	2031 (9.7)	864 (42.5)	564 (27.8)
Headaches	12 574 (59.9)	1929 (15.3)	3489 (27.7)
Sexual dysfunction	1423 (6.8)	535 (37.6)	389 (27.3)
Sleep issues or disorders	5981 (28.5)	1934 (32.3)	1578 (26.4)
Ulcer or gastrointestinal bleeding	1346 (6.4)	144 (10.7)	305 (22.7)
Back injury or back pain	10 494 (50.0)	2621 (25.0)	2361 (22.5)
Bowel or bladder abnormalities	2381 (11.3)	643 (27.0)	515 (21.6)
Skin abnormalities	2518 (12.0)	533 (21.2)	483 (19.2)
Kidney problems	1666 (7.9)	233 (14.0)	302 (18.1)
Substance use disorder (other than alcohol)	1200 (5.7)	266 (22.2)	190 (15.8)
Menstrual cramps (among female individuals, n = 11 171)	6994 (62.6)	759 (10.9)	1032 (14.8)
Dizzy or fainting spells	2857 (13.6)	654 (22.9)	389 (13.6)
Visual impairment (cataracts, glaucoma, macular or degeneration)	3517 (16.7)	865 (24.6)	452 (12.9)
Cancer	1716 (8.2)	113 (6.6)	221 (12.9)
Alcohol use disorder	1166 (5.6)	183 (15.7)	108 (9.3)
Nausea or vomiting	10 720 (51.0)	781 (7.3)	851 (7.9)
Liver or gallbladder problems	1958 (9.3)	153 (7.8)	130 (6.6)
Hernia	2195 (10.5)	275 (12.5)	120 (5.5)
Hearing impairment	2628 (12.5)	1129 (43.0)	117 (4.5)
Fracture	6150 (29.3)	204 (3.3)	207 (3.4)

### Data Quality

On the basis of the predetermined confidence ratings, the overall quality of the data was high, with only 2.5% of entries (1375 entries) noted as low confidence and excluded from the final analysis dataset. Of the remaining entries, 82.6% (44 773 entries) were rated as absolute confidence, 0.8% (455 entries) were rated as high confidence, and 16.6% (8992 entries) were rated as moderate confidence. The majority of the entries rated as moderate confidence were classified as such because more than 1 product was reported on one line of entry but in most instances the medications themselves were identified with certainty. The requirement for the quality review by the second investigator was met with 98.3% agreement of data lines. The 1.7% of data lines with disagreement were reconciled with full agreement between the principal investigator and the second investigator.

### Main Results

Past-7-day prevalence of any OTC or prescription medication was 62.3% (13 073 individuals) (Figure, panel A). Past-7-day use of 5 or more medications was reported by 3425 participants (16.3%), and 690 (3.3%) reported taking 10 or more medications. In general, medication usage was higher among female (7442 participants [66.6%]) than among male (5631 participants [57.3%]) participants, and the patterns of increasing use and number of medications with age were similar within the male and female cohorts. Past-7-day use was highest among those aged 65 years and older and was comparable between male (1541 participants [79.2%]) and female (2008 participants [81.9%]) individuals. The past-7-day prevalence of any OTC medication use (9657 participants [46.0%]) was similar to that of any prescription medication use (9719 participants [46.3%]) and illustrated a similar pattern of increased use with age among male and female individuals (Figure, panels B and C). Prevalence of prescription medication use increased more substantially with age compared with the increases in OTC medication use.

**Figure.**
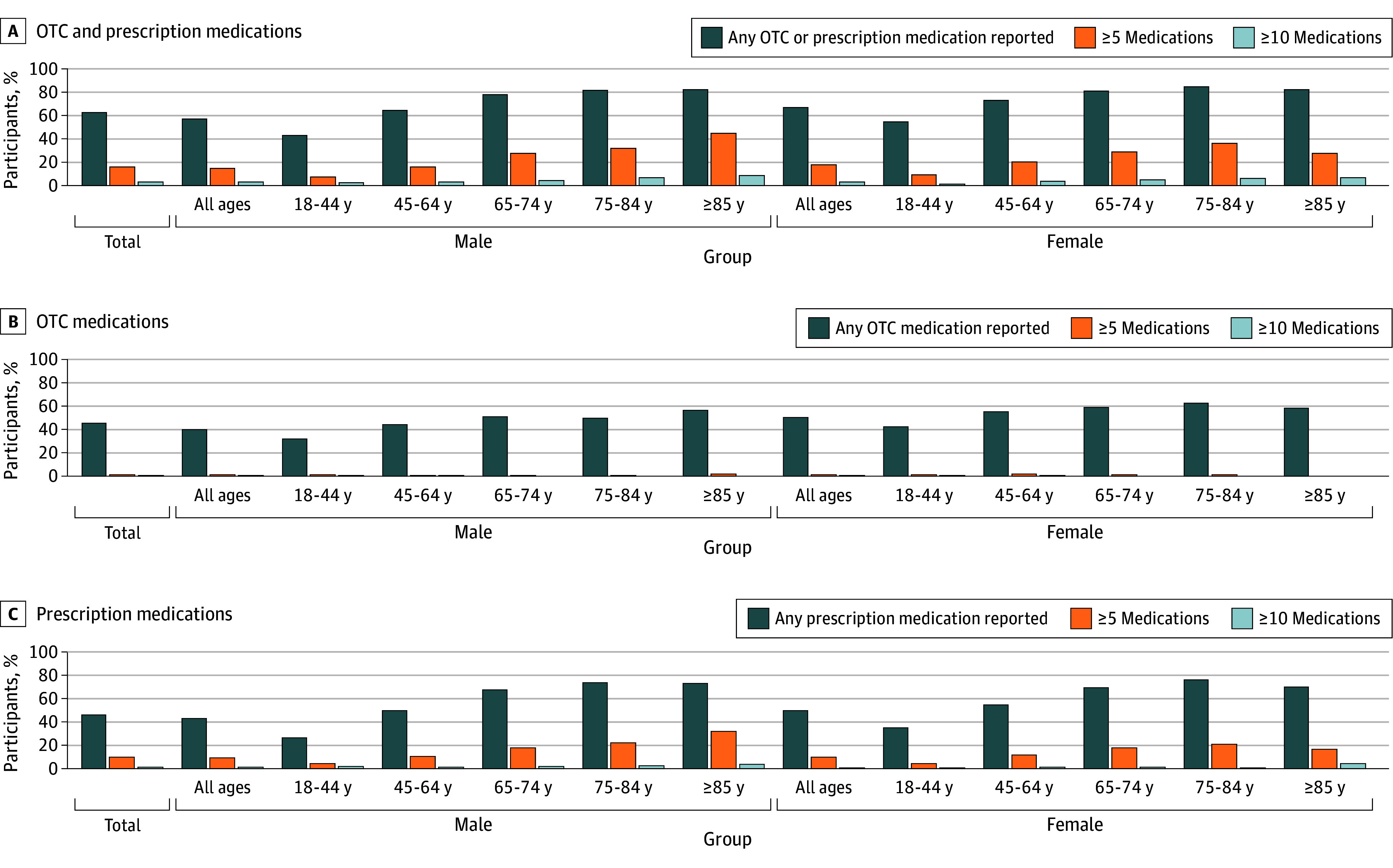
Bar Graphs of Prevalence of Past-7-Day Use of Medications by Sex and Age Graphs show data for any over-the-counter (OTC) or prescription medication by sex and age (A), any OTC medication by sex and age (B), and any prescription medication by sex and age (C).

Both overall and by sex, the most prevalent medications used in the past 7 days were acetaminophen (6184 individuals overall [29.4%]; 2452 male individuals, [24.9%]; 3732 female individuals [33.4%]), ibuprofen (4693 individuals overall [22.3%]; 1889 male individuals [19.2%]; 2804 female individuals [25.1%]), and aspirin (3323 individuals overall [15.8%]; 1705 male individuals [17.3%]; 1618 female individuals [14.5%]) (Table 3). For male individuals, the next most reported medications were atorvastatin (745 participants [7.6%]), naproxen (652 participants [6.6%]), lisinopril (633 participants [6.4%]), and metformin (501 participants [5.1%]). For female individuals, the next most reported medications were levothyroxine (835 participants [7.5%]), naproxen (802 participants [7.2%]), diphenhydramine (683 participants [6.1%]), and atorvastatin (597 participants [5.3%]). Six of the 10 most prevalent medications reported among all survey participants were available OTC. The most reported prescription medications overall were atorvastatin (1342 participants [6.4%]), lisinopril (1163 participants [5.5%]), levothyroxine (1086 participants [5.2%]), and amlodipine (965 participants [4.6%]). Patterns of prescription medication prevalence varied by sex and age (eTables 1 and 2 in Supplement 1).

**Table 3.** Top 50 Over-the-Counter and Prescription Medications Used: Past-7-Day Prevalence, Stratified by Sex

Rank	Medication^a^	Participants, No. (%)		
		Total (N = 21 000)	Male (n = 9829)	Female (n = 11 171)
1	Acetaminophen	6184 (29.4)	2452 (24.9)	3732 (33.4)
2	Ibuprofen	4693 (22.3)	1889 (19.2)	2804 (25.1)
3	Aspirin	3323 (15.8)	1705 (17.3)	1618 (14.5)
4	Naproxen	1454 (6.9)	652 (6.6)	802 (7.2)
5	Atorvastatin^b^	1342 (6.4)	745 (7.6)	597 (5.3)
6	Lisinopril^b^	1163 (5.5)	633 (6.4)	530 (4.7)
7	Levothyroxine^b^	1086 (5.2)	251 (2.6)	835 (7.5)
8	Diphenhydramine	1082 (5.2)	399 (4.1)	683 (6.1)
9	Omeprazole	969 (4.6)	432 (4.4)	537 (4.8)
10	Amlodipine^b^	965 (4.6)	487 (5.0)	478 (4.3)
11	Metformin^b^	934 (4.4)	501 (5.1)	433 (3.9)
12	Caffeine	928 (4.4)	364 (3.7)	564 (5.0)
13	Cetirizine	833 (4.0)	319 (3.2)	514 (4.6)
14	Hydrochlorothiazide^b^	828 (3.9)	367 (3.7)	461 (4.1)
15	Losartan^b^	813 (3.9)	378 (3.8)	435 (3.9)
16	Metoprolol^b^	756 (3.6)	369 (3.8)	387 (3.5)
17	Loratadine	606 (2.9)	237 (2.4)	369 (3.3)
18	Rosuvastatin^b^	542 (2.6)	273 (2.8)	269 (2.4)
19	Fluticasone	534 (2.5)	216 (2.2)	318 (2.8)
20	Sertraline^b^	524 (2.5)	156 (1.6)	368 (3.3)
21	Gabapentin^b^	521 (2.5)	191 (1.9)	330 (3.0)
22	Famotidine	505 (2.4)	199 (2.0)	306 (2.7)
23	Dextromethorphan	481 (2.3)	213 (2.2)	268 (2.4)
24	Calcium carbonate	478 (2.3)	176 (1.8)	302 (2.7)
25	Bupropion^b^	464 (2.2)	131 (1.3)	333 (3.0)
26	Phenylephrine	404 (1.9)	184 (1.9)	220 (2.0)
27	Albuterol^b^	381 (1.8)	143 (1.5)	238 (2.1)
28	Guaifenesin	380 (1.8)	155 (1.6)	225 (2.0)
29	Pantoprazole^b^	357 (1.7)	148 (1.5)	209 (1.9)
30	Estradiol^b^	351 (1.7)	42 (0.4)	309 (2.8)
31	Escitalopram^b^	344 (1.6)	94 (1.0)	250 (2.2)
32	Simvastatin^b^	312 (1.5)	157 (1.6)	155 (1.4)
33	Fexofenadine	310 (1.5)	117 (1.2)	193 (1.7)
34	Pseudoephedrine	300 (1.4)	124 (1.3)	176 (1.6)
35	Montelukast^b^	297 (1.4)	93 (0.9)	204 (1.8)
36	Amphetamine^b^	294 (1.4)	109 (1.1)	185 (1.7)
37	Duloxetine^b^	279 (1.3)	77 (0.8)	202 (1.8)
38	Fluoxetine^b^	266 (1.3)	87 (0.9)	179 (1.6)
39	Trazodone^b^	255 (1.2)	98 (1.0)	157 (1.4)
40	Tamsulosin^b^	244 (1.2)	236 (2.4)	8 (0.1)
41	Alprazolam^b^	243 (1.2)	84 (0.9)	159 (1.4)
42	Carvedilol^b^	241 (1.1)	126 (1.3)	115 (1.0)
43	Hydrocodone^b^	229 (1.1)	103 (1.0)	126 (1.1)
44	Insulin^b^	228 (1.1)	133 (1.4)	95 (0.9)
45	Venlafaxine^b^	218 (1.0)	69 (0.7)	149 (1.3)
46	Furosemide^b^	215 (1.0)	94 (1.0)	121 (1.1)
47	Dextroamphetamine^b^	210 (1.0)	66 (0.7)	144 (1.3)
48	Esomeprazole	203 (1.0)	92 (0.9)	111 (1.0)
49	Buspirone^b^	202 (1.0)	64 (0.7)	138 (1.2)
50	Apixaban^b^	200 (1.0)	111 (1.1)	89 (0.8)

^a^ Medications include single ingredient and fixed-dose combination medications and both over-the-counter and prescription medications, if available, that contain the active ingredient.

^b^ Drug was available by prescription only at the time of the study.

Compared with the Slone Survey,^5^ the top 3 ranked medications did not change, and the prevalence for those remaining in the top 20 were higher than previously reported except for aspirin (Table 4). Aspirin’s ranking remained as third most common, but the prevalence changed from 17.0% to 15.8%. Of the top 20 reported medications in this study, those previously only available as branded products but available as generic products during the current study increased in rank except for levothyroxine, which remained at seventh. Three of the 4 medications that became available OTC after the Slone Survey also increased in rank, with the fourth one (loratadine) remaining at 17th place. Four medications (omeprazole, amlodipine, metformin, and sertraline) were in the top 20 in the current study but not in the top 20 in the Slone Survey, and 3 medications (pseudoephedrine, conjugated estrogens, and estradiol) previously in the top 20 dropped to rankings of 34, 192, and 30, respectively.

**Table 4.** Comparison of Medication Prevalence Reported in Current Study vs Slone Survey

Medication^a^	Current study (2023-2024) (N = 21 000)		Slone survey (1998-1999) (n = 2590)^5^		Difference, Slone survey vs current study	
	Rank	Prevalence, %	Rank	Prevalence, %	Change in rank	Change in prevalence, %
Acetaminophen	1	29.4	1	23.0	No change	6.4
Ibuprofen	2	22.3	2	17.0	No change	5.3
Aspirin	3	15.8	3	17.0	No change	−1.2
Naproxen	4	6.9	12	3.5	8	3.4
Atorvastatin^b^^,^^c^	5	6.4	14	2.6	9	3.8
Lisinopril^b^	6	5.5	15	2.6	9	2.9
Levothyroxine^b^^,^^c^	7	5.2	7	4.2	No change	1.0
Diphenhdyramine	8	5.2	6	4.4	−2	0.8
Omeprazole^d^	9	4.6	22	2.1	13	2.5
Amlodipine^b^^,^^c^	10	4.6	26	1.7	16	2.9
Metformin^b^^,^^c^	11	4.4	33	1.4	22	3.0
Caffeine	12	4.4	9	3.9	−3	0.5
Cetirizine^d^	13	4.0	NA	NA	NA	NA
Hydrochlorothiazide^b^	14	3.9	10	3.7	−4	0.2
Losartan^b^^,^^c^	15	3.9	NA	NA	NA	NA
Metoprolol^b^	16	3.6	NA	NA	NA	NA
Loratadine^d^	17	2.9	17	2.5	No change	0.4
Rosuvastatin^b^	18	2.6	NA	NA	NA	NA
Fluticasone^d^	19	2.5	NA	NA	NA	NA
Sertraline^b^	20	2.5	37	1.2	17	1.3
Pseudoephedrine	34	1.4	4	8.1	−30	−6.7
Conjugated estrogens^b^	192	0.2	5	5.2	−187	−5.0
Estradiol^b^	30	1.7	8	4.2	−22	−2.5
Dextromethorphan	23	2.3	11	3.5	−12	−1.2
Chlorpheniramine	55	0.8	13	2.9	−42	−2.1
Medroxyprogesterone acetate^b^	320	0.1	16	2.6	−304	−2.5
Furosemide^b^	46	1.0	18	2.3	−28	−1.3
Phenylpropanolamine	NA	NA	19	2.3	NA	NA
Ranitidine	204	0.2	20	2.2	−184	−2.0

Abbreviation: NA, not applicable.

^a^ Medications include single ingredient and fixed-dose combination medications and both over-the-counter and prescription medications, if available, that contain the active ingredient.

^b^ Drug was available by prescription only at the time of the study.

^c^ Available as branded product only at the time of the Slone Survey but available as generic products during the current study period.

^d^ Prescription to over-the-counter switch: drug was available by prescription only at the time of the Slone Survey but available over-the-counter during the current study period.

## Discussion

In this survey study, nearly 2 of 3 participants (62.3%) reported use of any OTC or prescription medication in the past 7 days. As in previous reports,^2,5,6,11,14,16,20^ the use and number of medications used increased with age. The prevalence of OTC use (46.0%) was similar to that of prescription medications (46.3%), and 6 of the top 10 medications were available OTC, including the top 4 (acetaminophen, ibuprofen, aspirin, and naproxen). These prevalence estimates of actual usage demonstrate the substantial role of OTC medications in routine health management across demographic groups.

Although the current study methods varied from those of the 2002 Slone Survey, the top 3 medications (acetaminophen, ibuprofen, and aspirin) did not change from those reported 25 years prior. Among the Slone Survey top 20 medications, 11 remained in the top 20 and 9 dropped below. These observed changes in use prevalence align with changes in regulatory status and market availability, including medications that were available by prescription only during the Slone Survey but available OTC during the current study period, such as cetirizine, fluticasone, loratadine, and omeprazole, as well as medications that were previously only available as branded products but now have generic equivalents, including amlodipine, atorvastatin, levothyroxine, losartan, and metformin. Although causal inference cannot be made, these findings describe contemporary utilization patterns following regulatory changes.

The widespread increase in use of the most commonly reported medications is consistent with the overall increase in prescription medication usage in the US (up to 6.7 billion prescriptions in 2022 from 6.1 billion prescriptions in 2018).^27,28^ An aging population and increasing burdens of chronic disease, medicalization of aspects of daily living (such as obesity, substance use, hyperactivity, loneliness, and aging), ascendance of drugs to first-line treatment for an increasing array of medical conditions, and the development of new drugs or new uses for existing drugs have been identified as factors associated with the increase.^27^ The substantial decrease in use of some medications could potentially be associated with regulatory changes or related concerns that have arisen since the Slone Survey. For example, the prevalence of pseudoephedrine use decreased from 8.1% to 1.4% (rank fourth to 34th) following the Combat Methamphetamine Epidemic Act of 2005, which placed these medications behind the pharmacy counter, limited the purchase amount, and required additional documentation for purchase.^29^ Hormone therapies have dramatically expanded in the past 25 years, which may explain the substantial decreases in use of older medications like conjugated estrogens, estradiol, and medroxyprogesterone acetate. In 2005, the Food and Drug Administration (FDA) took steps to remove phenylpropanolamine from the US market after use was linked to increased risk of hemorrhagic stroke,^30^ and in 2020 the FDA announced a request to remove all ranitidine products from the market because of potential contamination with a probable human carcinogen.^31^

The accessibility and use of OTC medications have an added economic impact considering that 8.0% of the US population is uninsured and another 36.3% relies on public health care plans (eg, Medicare, Medicaid, or Veterans Administration).^32^ Compared with OTC medications, costs are typically higher for prescription medications and require health care practitioner visits, increasing the overall cost of care. Reducing the price of medications, improving the process through which prescription medications can be reclassified as OTC medications, and access to affordable life-saving medications were listed among the goals outlined in a 2025 Executive Order, Lowering Drug Prices by Once Again Putting Americans First.^33^ Presumably, this would include more novel switches in categories with no current OTC options. Estimation of public health outcomes associated with increased access to medications will require pharmacoeconomic studies. Currently, pharmacoeconomic studies of OTC medications are limited by lack of prevalence data, such as those presented in this study.

Understanding the prevalence of OTC medication use has relevance for regulatory science, public health surveillance, and pharmacoeconomic research. Prevalence estimates are commonly used to characterize baseline utilization, identify populations most likely to use specific medications, and inform postmarketing safety monitoring and modeling of potential public health impact. In this context, medication-specific prevalence data provide foundational information that may be incorporated into broader assessments of medication access and utilization, without implying appropriateness or effectiveness of any regulatory action.

Recent regulatory developments, including the FDA’s final rule outlining the application, labeling, and postmarketing reporting requirements for products seeking an additional condition for nonprescription use in December 2024,^34^ reflect ongoing interest in alternative mechanisms to support appropriate medication use without direct prescriber involvement. The current study does not evaluate the safety, effectiveness, or suitability of any medication for nonprescription use, nor does it assess outcomes of specific regulatory pathways. Rather, it provides contemporary, population-based prevalence estimates that may serve as contextual information for future evaluations of medication utilization and benefit-risk modeling.

### Limitations

This study has limitations that should be mentioned. First, it relied on self-report, recall of medication use, and researcher discretion in deciphering open-text fields. Several measures were taken to reduce potential reporting bias: (1) the primary outcome was past-7-day use, which is the time frame associated with the highest accuracy of recall^22,23^; (2) a stepwise approach to stimulating recall was used, first requiring free recall and then using prompts; (3) participants were instructed how to locate active ingredients on OTC and prescription labels and encouraged to gather their medications and use the internet or other sources as needed; and (4) autofill for product name and active ingredients was used to assist with accuracy of reporting, consistent spelling, and product recognition. Underreporting of some medications is possible as it was not feasible to ask medication-specific prompts for all medications as was done for the top 4, and the study was limited to oral prescription and OTC medications, excluding topical medications. If a medication was available both OTC and by prescription, it was assumed to be an OTC medication, potentially underestimating the prevalence of prescription medication use but avoiding the imprecision of assigning the medication to multiple medication categories (ie, OTC and prescription). Although the potential impact of seasonality was addressed with 3 survey time frames, a winter survey was not conducted.

## Conclusions

In this 2023 to 2024 study, nearly 2 of 3 US adults reported medication use in the past 7 days. Past-7-day prevalence and patterns of OTC and prescription medications demonstrate the magnitude of US reliance on these important therapies and highlight the importance of accessibility. Medication use data, both overall and medication specific, aids in regulatory decision-making and pharmacoeconomic evaluations and informs measures of benefit and risk. Continued monitoring is necessary to detect changes in medication use and to measure the impact of regulatory decisions or other changes that may affect medication access.

## Notes

Supplement 1. eTable 1. Top 50 over-the-counter (OTC) and prescription medications used by male participants, stratified by age eTable 2. Top 50 over-the-counter (OTC) and prescription medications used by female participants, stratified by age

Supplement 2. Data Sharing Statement
