# Supplementary material for: Prevalence of Over-the-Counter and Prescription Medication Use in the US
Source: JAMA Netw Open. 2026 Feb 16;9(2):e2559479. doi: 10.1001/jamanetworkopen.2025.59479 (PMC12910390; doi:10.1001/jamanetworkopen.2025.59479)
Supplement: Supplement 1. — eTable 1. Top 50 over-the-counter (OTC) and prescription medications used by male participants, stratified by age eTable 2. Top 50 over-the-counter (OTC) and prescription medications used by female participants, stratified by age [file jamanetwopen-e2559479-s001.pdf]

Supplemental Online Content

Green JL, Dailey-Govoni T, Kalidindi SD, Vosburg SK. Prevalence of over-the-counter and prescription medication use in the US. *JAMA Netw Open*. 2026;9(2):e2559479. doi:10.1001/jamanetworkopen.2025.59479

eTable 1. Top 50 over-the-counter (OTC) and prescription medications used by male participants, stratified by age

eTable 2. Top 50 over-the-counter (OTC) and prescription medications used by female participants, stratified by age

This supplemental material has been provided by the authors to give readers additional information about their work.

© 2026 Green JL et al. *JAMA Network Open*.

eTable 1. Top 50 over-the-counter (OTC) and prescription medications used by male participants, stratified by age

Rank	Medication[a]	Total (N=21,000)		Male											
				All Male (n=9,829)		18-44 y (n=4,560)		45-64 y (n=3,324)		65-74 y (n=1,253)		75-84 y (n=636)		85+ y (n=56)	
		n	%	n	%	n	%	n	%	n	%	n	%	n	%
1	acetaminophen	**6,184**	**29.4**	**2,452**	**24.9**	1,046	22.9	867	26.1	333	26.6	185	29.1	21	37.5
2	ibuprofen	**4,693**	**22.3**	**1,889**	**19.2**	899	19.7	689	20.7	212	16.9	86	13.5	3	5.4
3	aspirin	**3,323**	**15.8**	**1,705**	**17.3**	511	11.2	582	17.5	377	30.1	211	33.2	24	42.9
4	naproxen	**1,454**	**6.9**	**652**	**6.6**	274	6	242	7.3	89	7.1	43	6.8	4	7.1
5	*ATORVASTATIN*	**1,342**	**6.4**	**745**	**7.6**	46	1	315	9.5	239	19.1	130	20.4	15	26.8
6	*LISINOPRIL*	**1,163**	**5.5**	**633**	**6.4**	64	1.4	299	9	167	13.3	91	14.3	12	21.4
7	*LEVOTHYROXINE*	**1,086**	**5.2**	**251**	**2.6**	17	0.4	117	3.5	67	5.3	45	7.1	5	8.9
8	diphenhydramine	**1,082**	**5.2**	**399**	**4.1**	151	3.3	165	5	57	4.5	24	3.8	2	3.6
9	omeprazole	**969**	**4.6**	**432**	**4.4**	92	2	191	5.7	90	7.2	56	8.8	3	5.4
10	*AMLODIPINE*	**965**	**4.6**	**487**	**5**	51	1.1	189	5.7	148	11.8	85	13.4	14	25
11	*METFORMIN*	**934**	**4.4**	**501**	**5.1**	63	1.4	250	7.5	125	10	59	9.3	4	7.1
12	caffeine	**928**	**4.4**	**364**	**3.7**	188	4.1	121	3.6	30	2.4	21	3.3	4	7.1
13	cetirizine	**833**	**4**	**319**	**3.2**	146	3.2	91	2.7	56	4.5	24	3.8	2	3.6
14	*HYDROCHLOROTHIAZIDE*	**828**	**3.9**	**367**	**3.7**	44	1	164	4.9	89	7.1	61	9.6	9	16.1
15	*LOSARTAN*	**813**	**3.9**	**378**	**3.8**	26	0.6	150	4.5	121	9.7	74	11.6	7	12.5
16	*METOPROLOL*	**756**	**3.6**	**369**	**3.8**	35	0.8	132	4	121	9.7	71	11.2	10	17.9
17	loratadine	**606**	**2.9**	**237**	**2.4**	100	2.2	91	2.7	33	2.6	12	1.9	1	1.8
18	*ROSUVASTATIN*	**542**	**2.6**	**273**	**2.8**	13	0.3	121	3.6	85	6.8	51	8	3	5.4
19	fluticasone	**534**	**2.5**	**216**	**2.2**	60	1.3	82	2.5	46	3.7	24	3.8	4	7.1
20	*SERTRALINE*	**524**	**2.5**	**156**	**1.6**	61	1.3	68	2	14	1.1	13	2	0	0
21	*GABAPENTIN*	**521**	**2.5**	**191**	**1.9**	37	0.8	78	2.3	47	3.8	25	3.9	4	7.1
22	famotidine	**505**	**2.4**	**199**	**2**	69	1.5	77	2.3	28	2.2	24	3.8	1	1.8
23	dextromethorphan	**481**	**2.3**	**213**	**2.2**	107	2.3	63	1.9	23	1.8	17	2.7	3	5.4
24	calcium carbonate	**478**	**2.3**	**176**	**1.8**	51	1.1	87	2.6	19	1.5	18	2.8	1	1.8
25	*BUPROPION*	**464**	**2.2**	**131**	**1.3**	46	1	69	2.1	11	0.9	5	0.8	0	0
26	phenylephrine	**404**	**1.9**	**184**	**1.9**	108	2.4	54	1.6	14	1.1	7	1.1	1	1.8
27	*ALBUTEROL*	**381**	**1.8**	**143**	**1.5**	51	1.1	63	1.9	19	1.5	9	1.4	1	1.8
28	guaifenesin	**380**	**1.8**	**155**	**1.6**	76	1.7	43	1.3	17	1.4	17	2.7	2	3.6
29	*PANTOPRAZOLE*	**357**	**1.7**	**148**	**1.5**	23	0.5	58	1.7	43	3.4	20	3.1	4	7.1

© 2026 Green JL et al. *JAMA Network Open*.

Rank	Medication[a]	Total (N=21,000)		Male													
				All Male (n=9,829)		18-44 y (n=4,560)		45-64 y (n=3,324)		65-74 y (n=1,253)		75-84 y (n=636)		85+ y (n=56)	
		**n**	**%**	**n**	**%**	**n**	**%**	**n**	**%**	**n**	**%**	**n**	**%**	**n**	**%**
30	*ESTRADIOL*	**351**	**1.7**	**42**	**0.4**	31	0.7	6	0.2	2	0.2	3	0.5	0	0
31	*ESCITALOPRAM*	**344**	**1.6**	**94**	**1**	39	0.9	39	1.2	10	0.8	6	0.9	0	0
32	*SIMVASTATIN*	**312**	**1.5**	**157**	**1.6**	9	0.2	51	1.5	48	3.8	46	7.2	3	5.4
33	fexofenadine	**310**	**1.5**	**117**	**1.2**	62	1.4	34	1	13	1	8	1.3	0	0
34	pseudoephedrine	**300**	**1.4**	**124**	**1.3**	82	1.8	28	0.8	7	0.6	6	0.9	1	1.8
35	*MONTELUKAST*	**297**	**1.4**	**93**	**0.9**	22	0.5	47	1.4	17	1.4	6	0.9	1	1.8
36	*AMPHETAMINE*	**294**	**1.4**	**109**	**1.1**	76	1.7	30	0.9	2	0.2	1	0.2	0	0
37	*DULOXETINE*	**279**	**1.3**	**77**	**0.8**	15	0.3	39	1.2	18	1.4	5	0.8	0	0
38	*FLUOXETINE*	**266**	**1.3**	**87**	**0.9**	43	0.9	34	1	8	0.6	2	0.3	0	0
39	*TRAZODONE*	**255**	**1.2**	**98**	**1**	18	0.4	52	1.6	19	1.5	9	1.4	0	0
40	*TAMSULOSIN*	**244**	**1.2**	**236**	**2.4**	1	0	75	2.3	86	6.9	70	11	4	7.1
41	*ALPRAZOLAM*	**243**	**1.2**	**84**	**0.9**	30	0.7	31	0.9	14	1.1	8	1.3	1	1.8
42	*CARVEDILOL*	**241**	**1.1**	**126**	**1.3**	7	0.2	46	1.4	48	3.8	23	3.6	2	3.6
43	*HYDROCODONE*	**229**	**1.1**	**103**	**1**	34	0.7	47	1.4	17	1.4	3	0.5	2	3.6
44	*INSULIN*	**228**	**1.1**	**133**	**1.4**	17	0.4	58	1.7	38	3	20	3.1	0	0
45	*VENLAFAXINE*	**218**	**1**	**69**	**0.7**	20	0.4	38	1.1	6	0.5	5	0.8	0	0
46	*FUROSEMIDE*	**215**	**1**	**94**	**1**	8	0.2	27	0.8	33	2.6	21	3.3	5	8.9
47	*DEXTROAMPHETAMINE*	**210**	**1**	**66**	**0.7**	41	0.9	21	0.6	3	0.2	1	0.2	0	0
48	esomeprazole	**203**	**1**	**92**	**0.9**	21	0.5	47	1.4	16	1.3	8	1.3	0	0
49	*BUSPIRONE*	**202**	**1**	**64**	**0.7**	22	0.5	32	1	9	0.7	1	0.2	0	0
50	*APIXABAN*	**200**	**1**	**111**	**1.1**	7	0.2	26	0.8	42	3.4	29	4.6	7	12.5

[a] medications include single ingredient and fixed-dose combination medications and both OTC and prescription medications, if available, that contain the active ingredient; capitalized italic font indicates drug available by prescription only at the time of the study

© 2026 Green JL et al. *JAMA Network Open*.

eTable 2. Top 50 over-the-counter (OTC) and prescription medications used by female participants, stratified by age

Rank	Medication[a]	Total (N=21,000)		Female											
				All Female (n=11,171)		18-44 y (n=5,059)		45-64 y (n=3,660)		65-74 y (n= 1,723)		75-84 y (n=656)		85+ y (n=73)	
		n	%	n	%	n	%	n	%	n	%	n	%	n	%
1	acetaminophen	**6,184**	**29.4**	**3,732**	**33.4**	1,611	31.8	1,229	33.6	607	35.2	253	38.6	32	43.8
2	ibuprofen	**4,693**	**22.3**	**2,804**	**25.1**	1,399	27.7	960	26.2	335	19.4	101	15.4	9	12.3
3	aspirin	**3,323**	**15.8**	**1,618**	**14.5**	580	11.5	503	13.7	351	20.4	163	24.8	21	28.8
4	naproxen	**1,454**	**6.9**	**802**	**7.2**	319	6.3	300	8.2	132	7.7	44	6.7	7	9.6
5	*ATORVASTATIN*	**1,342**	**6.4**	**597**	**5.3**	31	0.6	226	6.2	231	13.4	104	15.9	5	6.8
6	*LISINOPRIL*	**1,163**	**5.5**	**530**	**4.7**	27	0.5	238	6.5	176	10.2	84	12.8	5	6.8
7	*LEVOTHYROXINE*	**1,086**	**5.2**	**835**	**7.5**	132	2.6	295	8.1	259	15	131	20	18	24.7
8	diphenhydramine	**1,082**	**5.2**	**683**	**6.1**	236	4.7	262	7.2	125	7.3	57	8.7	3	4.1
9	omeprazole	**969**	**4.6**	**537**	**4.8**	130	2.6	227	6.2	113	6.6	62	9.5	5	6.8
10	*AMLODIPINE*	**965**	**4.6**	**478**	**4.3**	42	0.8	171	4.7	172	10	84	12.8	9	12.3
11	*METFORMIN*	**934**	**4.4**	**433**	**3.9**	66	1.3	185	5.1	129	7.5	49	7.5	4	5.5
12	caffeine	**928**	**4.4**	**564**	**5**	281	5.6	185	5.1	69	4	26	4	3	4.1
13	cetirizine	**833**	**4**	**514**	**4.6**	197	3.9	187	5.1	91	5.3	37	5.6	2	2.7
14	*HYDROCHLOROTHIAZIDE*	**828**	**3.9**	**461**	**4.1**	33	0.7	178	4.9	162	9.4	79	12	9	12.3
15	*LOSARTAN*	**813**	**3.9**	**435**	**3.9**	27	0.5	159	4.3	167	9.7	72	11	10	13.7
16	*METOPROLOL*	**756**	**3.6**	**387**	**3.5**	30	0.6	146	4	138	8	65	9.9	8	11
17	loratadine	**606**	**2.9**	**369**	**3.3**	119	2.4	152	4.2	69	4	29	4.4	0	0
18	*ROSUVASTATIN*	**542**	**2.6**	**269**	**2.4**	14	0.3	102	2.8	110	6.4	39	5.9	4	5.5
19	fluticasone	**534**	**2.5**	**318**	**2.8**	83	1.6	124	3.4	80	4.6	28	4.3	3	4.1
20	*SERTRALINE*	**524**	**2.5**	**368**	**3.3**	176	3.5	112	3.1	53	3.1	27	4.1	0	0
21	*GABAPENTIN*	**521**	**2.5**	**330**	**3**	55	1.1	147	4	86	5	34	5.2	8	11
22	famotidine	**505**	**2.4**	**306**	**2.7**	74	1.5	125	3.4	68	3.9	37	5.6	2	2.7
23	dextromethorphan	**481**	**2.3**	**268**	**2.4**	120	2.4	94	2.6	37	2.1	17	2.6	0	0
24	calcium carbonate	**478**	**2.3**	**302**	**2.7**	101	2	118	3.2	64	3.7	17	2.6	2	2.7
25	*BUPROPION*	**464**	**2.2**	**333**	**3**	123	2.4	144	3.9	59	3.4	7	1.1	0	0
26	phenylephrine	**404**	**1.9**	**220**	**2**	95	1.9	85	2.3	27	1.6	13	2	0	0
27	*ALBUTEROL*	**381**	**1.8**	**238**	**2.1**	82	1.6	92	2.5	47	2.7	15	2.3	2	2.7
28	guaifenesin	**380**	**1.8**	**225**	**2**	82	1.6	84	2.3	40	2.3	19	2.9	0	0

© 2026 Green JL et al. *JAMA Network Open*.

Rank	Medication[a]	Total (N=21,000)		Female											
				All Female (n=11,171)		18-44 y (n=5,059)		45-64 y (n=3,660)		65-74 y (n= 1,723)		75-84 y (n=656)		85+ y (n=73)	
		n	%	n	%	n	%	n	%	n	%	n	%	n	%
29	*PANTOPRAZOLE*	**357**	**1.7**	**209**	**1.9**	25	0.5	87	2.4	62	3.6	33	5	2	2.7
30	*ESTRADIOL*	**351**	**1.7**	**309**	**2.8**	205	4.1	76	2.1	21	1.2	7	1.1	0	0
31	*ESCITALOPRAM*	**344**	**1.6**	**250**	**2.2**	139	2.7	66	1.8	33	1.9	12	1.8	0	0
32	*SIMVASTATIN*	**312**	**1.5**	**155**	**1.4**	4	0.1	35	1	62	3.6	47	7.2	7	9.6
33	fexofenadine	**310**	**1.5**	**193**	**1.7**	79	1.6	67	1.8	29	1.7	16	2.4	2	2.7
34	pseudoephedrine	**300**	**1.4**	**176**	**1.6**	75	1.5	69	1.9	21	1.2	10	1.5	1	1.4
35	*MONTELUKAST*	**297**	**1.4**	**204**	**1.8**	36	0.7	103	2.8	47	2.7	17	2.6	1	1.4
36	*AMPHETAMINE*	**294**	**1.4**	**185**	**1.7**	125	2.5	53	1.4	7	0.4	0	0	0	0
37	*DULOXETINE*	**279**	**1.3**	**202**	**1.8**	50	1	94	2.6	40	2.3	16	2.4	2	2.7
38	*FLUOXETINE*	**266**	**1.3**	**179**	**1.6**	74	1.5	61	1.7	39	2.3	5	0.8	0	0
39	*TRAZODONE*	**255**	**1.2**	**157**	**1.4**	44	0.9	76	2.1	28	1.6	8	1.2	1	1.4
40	*TAMSULOSIN*	**244**	**1.2**	**8**	**0.1**	3	0.1	2	0.1	2	0.1	1	0.2	0	0
41	*ALPRAZOLAM*	**243**	**1.2**	**159**	**1.4**	56	1.1	62	1.7	26	1.5	12	1.8	3	4.1
42	*CARVEDILOL*	**241**	**1.1**	**115**	**1**	5	0.1	42	1.1	43	2.5	22	3.4	3	4.1
43	*HYDROCODONE*	**229**	**1.1**	**126**	**1.1**	29	0.6	64	1.7	23	1.3	9	1.4	1	1.4
44	*INSULIN*	**228**	**1.1**	**95**	**0.9**	18	0.4	41	1.1	27	1.6	7	1.1	2	2.7
45	*VENLAFAXINE*	**218**	**1**	**149**	**1.3**	41	0.8	67	1.8	37	2.1	4	0.6	0	0
46	*FUROSEMIDE*	**215**	**1**	**121**	**1.1**	9	0.2	38	1	44	2.6	23	3.5	7	9.6
47	*DEXTROAMPHETAMINE*	**210**	**1**	**144**	**1.3**	103	2	38	1	3	0.2	0	0	0	0
48	esomeprazole	**203**	**1**	**111**	**1**	26	0.5	39	1.1	32	1.9	13	2	1	1.4
49	*BUSPIRONE*	**202**	**1**	**138**	**1.2**	63	1.2	49	1.3	15	0.9	9	1.4	2	2.7
50	*APIXABAN*	**200**	**1**	**89**	**0.8**	9	0.2	17	0.5	30	1.7	29	4.4	4	5.5

[a] medications include single ingredient and fixed-dose combination medications and both OTC and prescription medications, if available, that contain the active ingredient; capitalized italic font indicates drug available by prescription only at the time of the study

© 2026 Green JL et al. *JAMA Network Open*.
