# Supplementary material for: Prevalence of Over-the-Counter and Prescription Medication Use in the US
Source: JAMA Netw Open. 2026 Feb 16;9(2):e2559479. doi: 10.1001/jamanetworkopen.2025.59479 (PMC12910390; doi:10.1001/jamanetworkopen.2025.59479)
Supplement: Supplement 2. — Data Sharing Statement [file jamanetwopen-e2559479-s002.pdf]

Data Sharing Statement

Green. Prevalence of Over-the-Counter and Prescription Medication Use in the US. *JAMA Netw Open*. Published February 16, 2026. doi:10.1001/jamanetworkopen.2025.59479

Data
Data available: No
